# Endothelial ROBO4 suppresses PTGS2/COX-2 expression and inflammatory diseases

**DOI:** 10.1038/s42003-024-06317-z

**Published:** 2024-05-18

**Authors:** Masato Tanaka, Keisuke Shirakura, Yui Takayama, Miki Μatsui, Yukio Watanabe, Takuya Yamamoto, Junya Takahashi, Shota Tanaka, Nobumasa Hino, Takefumi Doi, Masanori Obana, Yasushi Fujio, Kazuo Takayama, Yoshiaki Okada

**Affiliations:** 1 Graduate School of Pharmaceutical Sciences, Osaka University, Osaka, Japan; 2 Center for iPS Cell Research and Application (CiRA), Kyoto University, Kyoto, Japan; 3 Medical-risk Avoidance based on iPS Cells Team, RIKEN Center for Advanced Intelligence Project (AIP), Kyoto, Japan; 4 Institute for the Advanced Study of Human Biology (WPI-ASHBi), Kyoto University, Kyoto, Japan; 5 Center for Infectious Disease Education and Research (CiDER), Osaka University, Osaka, Japan; 6 AMED-CREST, Japan Agency for Medical Research and Development (AMED), Tokyo, Japan

**Keywords:** Cell signalling, Molecular medicine

## Abstract

Accumulating evidence suggests that endothelial cells can be useful therapeutic targets. One of the potential targets is an endothelial cell-specific protein, Roundabout4 (ROBO4). ROBO4 has been shown to ameliorate multiple diseases in mice, including infectious diseases and sepsis. However, its mechanisms are not fully understood. In this study, using RNA-seq analysis, we found that ROBO4 downregulates prostaglandin-endoperoxide synthase 2 (PTGS2), which encodes cyclooxygenase-2. Mechanistic analysis reveals that ROBO4 interacts with IQ motif-containing GTPase-activating protein 1 (IQGAP1) and TNF receptor-associated factor 7 (TRAF7), a ubiquitin E3 ligase. In this complex, ROBO4 enhances IQGAP1 ubiquitination through TRAF7, inhibits prolonged RAC1 activation, and decreases PTGS2 expression in inflammatory endothelial cells. In addition, Robo4-deficiency in mice exacerbates PTGS2-associated inflammatory diseases, including arthritis, edema, and pain. Thus, we reveal the molecular mechanism by which ROBO4 suppresses the inflammatory response and vascular hyperpermeability, highlighting its potential as a promising therapeutic target for inflammatory diseases.

## Abstract (web summary)

ROBO4 suppresses PTGS2/COX-2 expression by inhibiting prolonged RAC1 activation in endothelial cells, ameliorating inflammatory diseases in mice; these endothelial molecules represent potential therapeutic targets for inflammatory diseases.

## Introduction

Endothelial cells (ECs) lining the inner surface of blood vessels play essential roles in regulating the inflammatory response. During physiological inflammation, ECs regulate the migration and activity of immune cells by inducing inflammatory genes, secreting cytokines and other proteins^1,2^, and increasing vascular permeability^3,4^. Since this EC-mediated inflammatory response also contributes to the development of inflammatory diseases, ECs as well as immune cells are considered to be an important therapeutic target. In fact, EC-specific inhibition of inflammatory signaling or enhancement of EC-specific proteins ameliorates severe infectious diseases, atherosclerosis, and arthritis^5–8^. Therefore, understanding the molecular mechanisms underlying inflammatory response in ECs is crucial for developing therapeutic strategies.

Cyclooxygenase-2 (COX-2/PTGS2), encoded by prostaglandin-endoperoxide synthase 2 (*PTGS2*), regulates inflammation and homeostasis by synthesizing lipid mediators such as prostanoids. PTGS2 expression is induced by inflammatory stimuli in various cell types, including endothelial, epithelial, and immune cells. Endothelial PTGS2 contributes to the development of inflammatory diseases, including arthritis and tumors, by enhancing fever, pain, and angiogenesis^9–14^. Although the contribution of endothelial PTGS2 to inflammatory and other diseases is well-known, the mechanisms regulating PTGS2 expression in ECs are not fully understood.

Roundabout4 (ROBO4) is an EC-specific membrane protein that regulates endothelial permeability, cytokine production, and angiogenesis in pathological conditions^1,15–17^. Robo4 ameliorates several diseases in mice, including infectious diseases, sepsis, retinopathy, and tumors^15,16,18,19^. In the retinopathy and tumor models, Robo4 suppresses angiogenesis by regulating the activity of vascular endothelial growth factor receptor 2, the small GTPase RAC1, and other proteins^16,17,20,21^. In endotoxemia and infectious disease models, Robo4 suppresses vascular hyperpermeability and mortality induced by lipopolysaccharide, *Escherichia coli*, influenza virus, and SARS-CoV-2^15,19,22^, indicating that ROBO4 is a promising therapeutic target for infectious and inflammatory diseases. In the endotoxemia models, ROBO4 was shown to suppress vascular hyperpermeability by interacting with TNF receptor-associated factor 7 (TRAF7), a ubiquitin E3 ligase^15^. However, the detailed mechanisms whereby ROBO4 suppresses vascular permeability remain unknown. It is also unclear whether ROBO4 suppresses diseases through other mechanisms.

In this study, we investigated ROBO4-regulated genes in inflammatory ECs using RNA-seq analysis and identified PTGS2, a target of ROBO4. We demonstrated that ROBO4 suppresses endothelial PTGS2 expression and hyperpermeability by inhibiting prolonged RAC1 activation via a newly identified protein complex. Moreover, we revealed that ROBO4 partially suppresses PTGS2-associated inflammatory diseases, including arthritis, edema, and pain in mouse models.

## Methods

### Cell culture

Human umbilical vein endothelial cells (HUVECs) (Lonza) were cultured in EGM-2-MV medium (Lonza). Human embryonic kidney cells (HEK293) and African Green monkey SV40-transfected kidney fibroblast cells (COS-7 cells) were cultured in Dulbecco’s modified Eagle’s medium (Nacalai Tesque) supplemented with 10% fetal bovine serum (FBS), 100 IU/mL penicillin, and 100 μg/mL streptomycin. All cells were cultured at 37 °C in an atmosphere containing 5% CO_2_.

### Small interfering RNA-mediated gene knockdown

siRNAs for *ROBO4* (SI03066896), *CTNNB1* (SI00029750), *IQGAP1* (SI02655268), and the controls (AllStars negative control) were purchased from Qiagen. The siRNA sequences are listed in Table S1. For knockdown experiments, HUVECs (1–8 × 10^5^ cells) were transfected with siRNA (12.5–100 pmol) using Lipofectamine RNAiMAX (Thermo Fisher Scientific).

### Treatment with inhibitors and TNF

HUVECs were transfected with siRNAs and cultured in EBM-2 medium (Lonza) containing 0.5% FBS for 20 h. The resulting cells were pretreated with or without inhibitors for AP-1 (SR 11302, Cayman Chemical; 1 μM for 1 h), RAC1 (NSC23766, Tocris Bioscience; 100 μM for 20 h), or JNK (SP600125, Sigma-Aldrich; 10 μM for 30 min) and then stimulated with TNF (1 µg/mL; FUJIFILM Wako Pure Chemicals) for 6 h.

### RNA sequencing

HUVECs (2 × 10^5^ cells) were seeded in 6-well plates and transfected with siRNA for *ROBO4*. The cells were then treated with TNF and lysed with the buffer RLT (Qiagen) directly on the plates, and total RNA was extracted using the RNeasy Mini Kit (Qiagen). RNA integrity was assessed using the 2100 Bioanalyzer (Agilent Technologies). The library was prepared with 100 ng of total RNA using a TruSeq Stranded mRNA sample preparation kit (Illumina), according to the manufacturer’s instructions. Sequencing was performed using Illumina NextSeq550. FASTQ files were generated using bcl2fastq-2.20. Adapter sequences and low-quality bases were trimmed from the raw reads using Cutadapt ver v3.4^23^. Trimmed reads were mapped to the human reference genome sequences (hg38) using STAR version 2.7.9a^24^ (https://software.cqls.oregonstate.edu/updates/star-2.7.9a/) with the GENCODE (release 36, GRCh38.p13)^25^ GTF file (https://www.gencodegenes.org/human/release_36.html). Raw counts were calculated using htseq-count ver. 0.13.5^26^, using a GENCODE GTF file. Gene expression levels were determined as transcripts per kilobase million (TPM) using DESeq2 v1.30.1^27^ (https://bioconductor.org/packages/release/bioc/html/DESeq2.html). Raw data from this study were submitted under the Gene Expression Omnibus (GEO) (accession number GSE231460).

### Quantitative reverse transcription-polymerase chain reaction (RT-PCR)

Total RNA was prepared from the cells using the RNeasy Mini Kit (Qiagen). Total RNA was reverse transcribed using Superscript VILO Master Mix (Thermo Fisher Scientific). Real-time RT-PCR was performed using the QuantiTect SYBR Green PCR Kit (Qiagen) and specific primers (Table S2) on a CFX384 Touch Real-Time PCR Detection System (Bio-Rad). Copy numbers were calculated from standard curves constructed using known amounts of plasmids containing target sequences. Expression levels were normalized to those of *GAPDH*.

### Preparation of nuclear and cytoplasmic proteins

HUVECs were treated with siRNA for 24 h and cultured in EBM-2 medium containing 0.5% FBS for 20 h. The cells were stimulated with TNF (1 µg/mL) for 6 h and then used for preparation of nuclear and cytoplasmic proteins using a Qproteome cell compartment kit (Qiagen). Each cellular fraction (1 μg) was analyzed separately by western blotting.

### Immunoprecipitation and mass spectrometry

Mass-spectrometry-based analysis of Robo4 binding proteins has been reported previously^15^. HUVECs were infected with adenoviral vectors encoding ROBO4-FLAG or *Aequorea coerulescens* green fluorescent protein (AcGFP) and incubated for 36 h. The resulting cells were lysed in lysis buffer (50 mM Tris-HCl [pH 7.4], 150 mM NaCl, 1% Triton X-100, 0.5% sodium deoxycholate, 1 mM EDTA, and a protease inhibitor cocktail [Roche]). The resulting lysates were used for immunoprecipitation using a FLAG immunoprecipitation kit (Sigma-Aldrich). The precipitated proteins were digested with trypsin and analyzed by liquid chromatography-tandem mass spectrometry using an Ultimate 3000 and Q Exactive (Thermo Fisher Scientific). The obtained data were analyzed using Mascot Software (Matrix Science) to identify proteins that were specifically included in the ROBO4-FLAG sample but not in the AcGFP sample.

For the analysis of the interaction between IQGAP1 and ROBO4 or TRAF7, COS-7, or HEK293 cells were transfected with expression vectors (3 μg of each) using Lipofectamine2000 (Thermo Fisher Scientific) and cultured for 24 h. The resulting cells were lysed in lysis buffer and subjected to immunoprecipitation using a FLAG immunoprecipitation kit and an anti-IQGAP1 antibody (C-9 AC, Santa Cruz Biotechnology). The precipitated proteins were analyzed by western blotting.

To analyze IQGAP1 ubiquitination and the interaction between IQGAP1 and RAC1, HUVECs (2 × 10^5^ cells) were seeded in each well of a 24-well plate and transfected with siRNAs, followed by incubation for 2 days. These cells were treated with MG132 (10 μΜ for 1 h; Fuji Film) and stimulated with TNF (1 μg/mL for 4 h). HEK293 cells were transfected with expression vectors (3 μg of each) encoding HA-ubiquitin, ROBO4, and TRAF7 using Lipofectamine2000 and cultured for 20 h. The cells were then treated with MG132 (10 μM for 4 h). The resulting HUVECs and HEK293 cells were subjected to immunoprecipitation assays using an anti-IQGAP1 antibody (C-9 AC, Santa Cruz Biotechnology) or anti-RAC1 antibody (23A8, Merck Millipore). The precipitated proteins were analyzed by western blotting.

### Western blotting

Cell lysates and immunoprecipitated samples were separated using sodium dodecyl sulfate-polyacrylamide gel electrophoresis and transferred onto polyvinylidene fluoride membranes. Membranes were blocked with 3% skim milk or 5% bovine serum albumin in Tris-buffered saline containing Tween 20. The resulting membranes were then incubated with primary antibodies against β-catenin/catenin beta-1 (E-5, Santa Cruz Biotechnology), PTGS2/COX-2 (D5H5, Cell Signaling Technology), FLAG tag (M2, Sigma-Aldrich), GAPDH (1E6D9, Proteintech), HA tag (3F10, Roche), IQGAP1 (H109, Santa Cruz Biotechnology), JNK (#9252, Cell Signaling Technology), phospho-SAPK/JNK (81E11, Cell Signaling Technology), Lamin B1 (3C10G12, Proteintech), Myc tag (9B11, Cell Signaling Technology), ROBO4 (AF2366, R&D Systems), TRAF7 (H-300, Santa Cruz Biotechnology), mono- and poly-ubiquitinylated conjugates (FK2, Enzo Life Sciences), and RAC1 (PA1-091, Thermo Fisher Scientific) and secondary antibodies conjugated with horseradish peroxidase. Immunoreactive bands were detected using an ImageQuant LAS4010 system (GE Healthcare). The primary antibodies used are listed in Table S3.

### Quantification of active RAC1

HUVECs were transfected with siRNAs and cultured for 2 days. The cells were then cultured in EBM-2 containing 0.5% FBS for 20 h and stimulated with TNF (1 µg/mL) for 0.25–8 h. The cells were lysed on the culture plates, and then the levels of active RAC1 in the cells were measured using a RAC1 G-LISA activation assay kit (cytoskeleton).

### Measurement of transendothelial electrical resistance (TEER)

HUVECs (4 × 10^4^ cells) were seeded in cell culture inserts with a pore size of 0.4 µm (BD Falcon), treated with siRNAs (2.5  pmol), and incubated for 48 h. These cells were pretreated with NSC23766 (100 μM for 1 h) and TNF (1 μg/mL). TEER was measured using a cellZScope (NanoAnalytics). The TEER value was calculated using the following formula: (resistance of experimental wells − resistance of blank wells) × 0.32 (the membrane area of the cell culture insert).

### Immunofluorescence staining

HUVECs on gelatin-coated cover glass were treated with siRNA and incubated for 48 h. These cells were pretreated with NSC23766 (100 μM for 1 h) and stimulated with TNF (1 μg/mL for 8 h). The resulting cells were fixed with 4% paraformaldehyde for 10 min, permeabilized with 0.3% TritonX-100 at 25 °C for 5 min, and blocked with 1% bovine serum albumin for 30 min. The cells were then incubated with an anti-cadherin-5 antibody (F-8; Santa Cruz Biotechnology) at 4 °C overnight and a secondary antibody conjugated with Alexa Fluor 555 (A21424; Thermo Fisher Scientific) at 25 °C for 30 min. The cells were mounted with Vectashield mounting medium containing DAPI (Vector Laboratories) and analyzed using BZ-X700 (KEYENCE).

### Quantification of cadherin-5 discontinuity

Cadherin-5 discontinuity was quantified using ImageJ/Fiji^28^. To obtain an outline of each cell, “subtract background at rolling = 20” was applied to the cadherin-5 channel, followed by the application of the “Gaussian blue at Sigma = 2.0” filter twice and the “sharpen” filter, subsequently, the image was converted to a binary image using AutoThreshold set to “Huang”. The binary image underwent “dilution” twice, followed by manual correction using the line tool and “skeleton”. Twenty-five cell outlines were randomly selected per image. To detect discontinuous cadherin-5 structure, the filter “subtract background at rolling = 20” was applied to the cadherin-5 channel, which was then converted to a binary image with a threshold set to 21–255. The signal on the complete cell outlines was measured using a “plot profile”. Finally, to calculate the ratio of cadherin-5 discontinuity, the number of pixels with an intensity 0 was expressed as a percentage of the total pixels of the cell outlines (ratio of cadherin-5 discontinuity %).

### Expression vectors

Preparation of the adenoviral vector for ROBO4-FLAG and expression vectors for ROBO4 (pcDNA3-ROBO4), ROBO4-FLAG (pHMEF5-ROBO4-FLAG), and TRAF7 (pcDNA3-TRAF7) has been reported^15^. To prepare expression vectors for IQGAP1 (pcDNA4-IQGAP1-Myc) and HA-ubiquitin (pOrip-HA-ubiquitin), DNA fragments encoding IQGAP1-myc and HA-ubiquirepotin were amplified by PCR using HUVEC cDNA and specific primers (Table S1). These fragments were inserted into pcDNA4 (Thermo Fisher Scientific) and pOriP vectors^29^.

### Mouse inflammatory disease models

The animal studies were approved by the ethics committees of Charles River Laboratories Japan, Inc. (approval numbers 1358, 1359, and 1579) and Graduate School of Pharmaceutical Sciences, Osaka University (approval number Douyaku 28-15), and performed by Charles River Laboratories Japan, Inc. We have complied with all relevant ethical regulations for animal use. For the collagen-induced arthritis model, male *Robo4*^+/+^ and *Robo4*^−/^^−^ mice^1,15^ (male, 11–13-weeks-old) were subcutaneously injected with 25 μL of 0.3% type II collagen (Collagen Research Center) emulsified in Freund’s complete adjuvant (Sigma-Aldrich). After 3 weeks, collagen was injected again; the arthritis score and body weight were measured. The arthritis score (from 0 to 4) was determined by clinically scoring the joint inflammation in each paw (Table S4). For the carrageenan-induced paw edema model^30^, *Robo4*^+/+^ and *Robo4*^−^^/^^−^ mice (male, 11–13-weeks-old) were injected with a 1% carrageenan solution (30 μL/mouse). After 4 h, paw volume was measured using a plethysmometer MK-101CMP (Muromachi Kikai). For the acetic acid-induced pain model^31–34^, *Robo4*^+/+^ and *Robo4*^−^^/^^−^ mice (male, 11–13-weeks-old) were intraperitoneally injected with 0.6% acetic acid solution (10 mL/kg). The number of writhing reactions, characterized by contraction of the abdomen and extension of the trunk and hind limbs, was counted for each mouse over a period of 30 min.

### Statistics and reproducibility

Data are expressed as mean ± standard error of at least three independent experiments. *P* values were calculated using Prism10 (GraphPad Inc.) (Table S5). The statistical significance of the differences was determined using the tests shown in the figure legends. Statistical significance was set at *P* < 0.05. Sample size and numbers are indicated in each figure legend.

### Reporting summary

Further information on research design is available in the Nature Portfolio Reporting Summary linked to this article.

## Results

### Investigation of ROBO4-regulated genes in ECs under inflammation

**Fig. 1:**
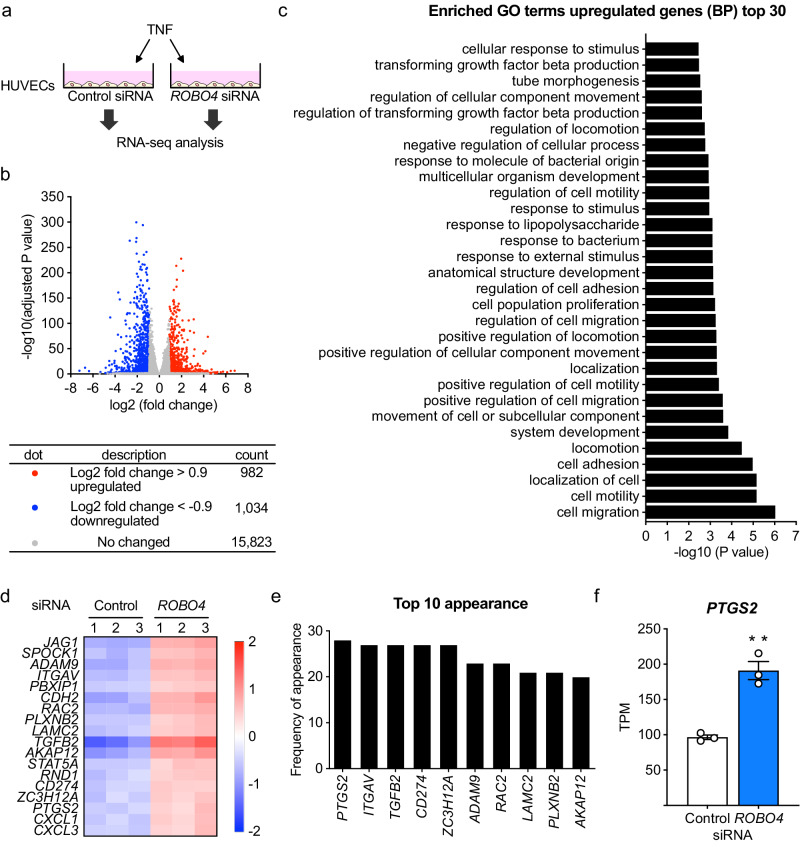
Identification of ROBO4-regulated genes in TNF-treated ECs by RNA-seq analysis. **a** RNA sequencing analysis was performed using human umbilical vein ECs (HUVECs) treated with TNF (1 μg/mL) and siRNA. Three samples for Control or *ROBO4* siRNA were prepared and used for RNA-seq analysis. **b** A volcano plot of differentially expressed genes between TNF-treated HUVECs transfected with control or *ROBO4* siRNA (log2 fold-change > 0.9, adjusted *P* value (*P*_adj_) < 0.05). **c** Gene ontology (GO) enrichment analysis of control siRNA-transfected HUVECs versus *ROBO4* siRNA-transfected HUVECs. **d** A heatmap of the 18 upregulated genes in the “cell migration” category in HUVECs transfected with *ROBO4* siRNA as compared to control siRNA. **e** The frequency of appearance of the genes included in the “cell migration” category in the top 30 GO terms is shown in (**c**). **f** TPM value of *PTGS2* (the gene encoding COX-2) in HUVECs transfected with Control or *ROBO4* siRNA. Data are expressed as the mean ± standard error of the mean. **P < 0.01; *P* values were calculated using an unpaired *t*-test.

To identify the ROBO4-regulated genes in inflammatory ECs, we performed RNA-seq analysis using TNF- and siRNA-treated HUVECs, which show a high expression of endogenous ROBO4 (Fig. 1a and Supplementary Fig. S1). *ROBO4* siRNA transfection upregulated 982 genes and downregulated 1034 genes by Log2 0.9-fold or more in HUVECs (Fig. 1b). Enrichment analysis showed an increase in gene expression in categories, such as “cell migration,” “cell motility,” and “localization of cells.” (Fig. 1c). As ROBO4 has been shown to regulate endothelial migration^35^, we focused on 18 differentially expressed genes in the “cell migration” category (Fig. 1d). Among these, we identified *PTGS2* (the gene encoding COX-2), which was upregulated by *ROBO4* siRNA transfection and appeared most frequently in the top 30 categories (28 times), as an inflammatory-related gene (Fig. 1e). In the presence of TNF, *PTGS2* expression in *ROBO4* siRNA-transfected ECs was significantly higher than that in control siRNA-transfected ECs (Fig. 1f). Taken together, these results indicated that *PTGS2* is a potential ROBO4-regulated gene in ECs under inflammatory conditions.

### ROBO4 suppresses PTGS2 expression by inhibiting AP-1 and catenin beta-1

**Fig. 2:**
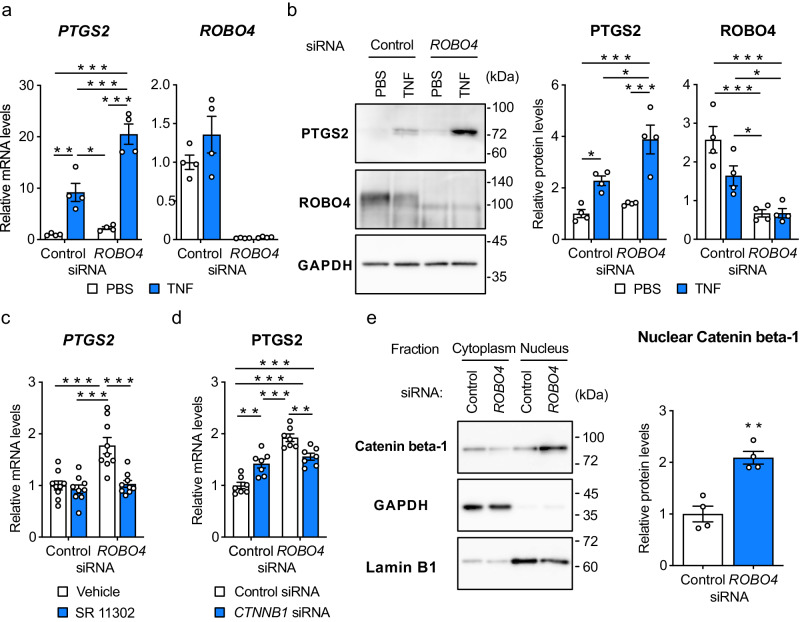
ROBO4 suppresses PTGS2 expression by inhibiting AP-1 and catenin beta-1. **a**, **b** Effect of ROBO4 on TNF-induced PTGS2 expression in ECs. HUVECs were treated with siRNA and TNF, and expression of *PTGS2*, *Robo4*, and *GAPDH* mRNA and proteins were measured by quantitative reverse transcription polymerase chain reaction (qRT-PCR) (**a**) and immunoblotting (**b**), respectively. PTGS2 and Robo4 levels were normalized to GAPDH levels (*n* = 4). **c**, **d** *PTGS2* expression in HUVECs treated with siRNA, TNF, and AP-1 inhibitor (SR 11302) (**c**) or *CTNNB1* siRNA (**d**) (*n* = 9 or 7, respectively). *PTGS2* and *GAPDH* mRNA levels were measured by qRT-PCR. **e** Effect of ROBO4 on subcellular localization of catenin beta-1. Cytoplasmic and nuclear protein extracts were prepared from HUVECs treated with siRNA and TNF, and analyzed for catenin beta-1, GAPDH, and Lamin B1 expression by western blotting. Expression levels of catenin beta-1, GAPDH and Lamin B1 were quantified using the ImageJ software (*n* = 4). Relative nuclear catenin beta-1 levels were calculated by normalizing to Lamin B1 levels. Data are expressed as the mean ± standard error of the mean (**a**–**e**). *P < 0.05, **P < 0.01, and ***P < 0.001, *P* values were calculated with two-way analysis of variance followed by Tukey’s test (**a**–**d**) or Welch’s *t*-test (**e**). Non-specified *P* values in the graph are not significant.

To confirm that ROBO4 regulates PTGS2 expression in inflammatory ECs, we analyzed *PTGS2* expression levels using quantitative polymerase chain reaction (qPCR) and immunoblotting (Fig. 2a, b). In the qPCR analysis, TNF treatment for 6 h increased *PTGS2* mRNA expression in ECs treated with control siRNA. *ROBO4* siRNA further increased the induced PTGS2 expression (Fig. 2a and Supplementary Fig. S2a). Similarly, immunoblotting analysis demonstrated that *ROBO4* siRNA enhanced TNF-induced PTGS2 expression (Fig. 2b). *ROBO4* siRNA also enhanced *PTGS2* expression by other inflammatory mediators, including IL1B and lipopolysaccharides (Supplementary Fig. S2b, c). These results indicated that ROBO4 suppresses PTGS2 expression in ECs treated with inflammatory mediators, including TNF.

We then investigated the mechanism whereby ROBO4 regulates PTGS2 expression. PTGS2 expression is known to be regulated by activator protein-1 (AP-1)^36,37^, which cooperatively regulates gene expression with catenin beta-1 (also known as β-catenin)^38^. Therefore, we investigated the effects of AP-1 and catenin beta-1 on PTGS2 expression. The AP-1 inhibitor SR 11302 suppressed *PTGS2* upregulation induced by *ROBO4* siRNA (Fig. 2c). Similarly, *CTNNB1* siRNA inhibited *PTGS2* upregulation induced by *ROBO4* siRNA (Fig. 2d). Additionally, *ROBO4* siRNA increased catenin beta-1 expression in the nucleus (Fig. 2e). Taken together, these results indicated that ROBO4 suppresses PTGS2 expression by inhibiting AP-1 and reducing nuclear catenin beta-1 levels.

### ROBO4 suppresses PTGS2 expression by inhibiting RAC1-JNK signaling

**Fig. 3:**
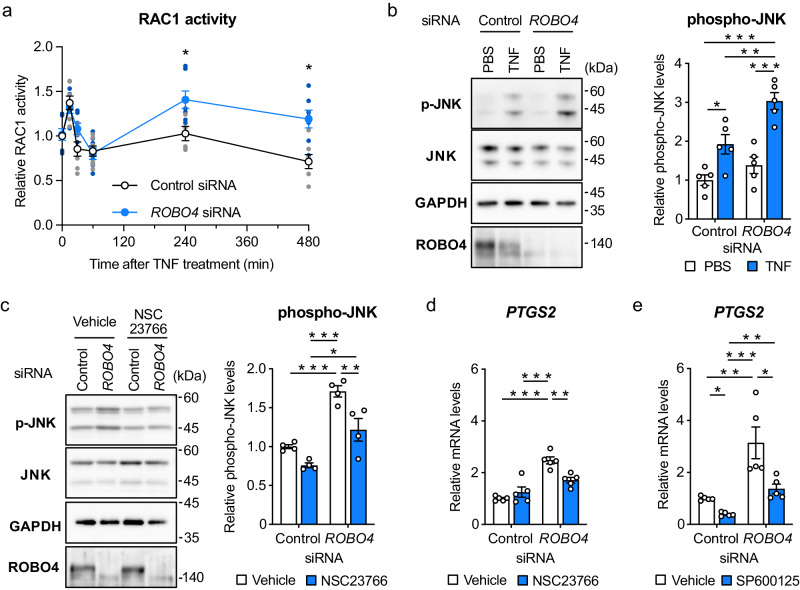
ROBO4 suppresses PTGS2 expression by inhibiting RAC1-JNK signaling. **a** Effect of ROBO4 on active RAC1 in ECs. Cell extracts were prepared from HUVECs treated with siRNA and TNF and used for measurements of active RAC1 using a G-LISA kit (*n* = 7). **b**, **c** Regulation of JNK phosphorylation by ROBO4 and RAC1. HUVECs were transfected with siRNA and treated with TNF in the presence or absence of pretreatment with NSC23766 (100 μM). Expression levels of p-JNK, JNK, GAPDH, and ROBO4 in the cells were analyzed by western blotting. Relative p-JNK levels were quantified using ImageJ software and calculated by normalizing with JNK levels (*n* = 5 (**b**); *n* = 4 (**c**)). **d**, **e** Contribution of RAC1 and JNK to *ROBO4* knockdown-induced *PTGS2* upregulation. HUVECs were transfected with siRNA, pretreated with NSC23766 (100 μΜ) or SP600125 (10 μM), and treated with TNF. Expression of *PTGS2* and *GAPDH* were measured by qRT-PCR (*n* = 5). Data are expressed as the mean ± standard error of the mean (**a**–**e**). *P < 0.05, **P < 0.01, and ***P < 0.001, *P* values were calculated with a two-way analysis of variance followed by Bonferroni’s test between control and *ROBO4* siRNA groups at each time point (**a**) or Tukey’s test (**b**–**d**). Non-specified *P* values in the graph are not significant.

Previous studies have demonstrated that c-Jun, a component of AP-1, and catenin beta-1 are involved in the same protein complex regulated by JNK^38^, and that RAC1-JNK signaling promotes the nuclear translocation of catenin beta-1^7,39,40^. Additionally, ROBO4 was shown to regulate RAC1 activity^16,21^. Thus, we hypothesized that ROBO4 suppresses PTGS2 expression by inhibiting RAC1-JNK signaling, which activates AP-1 and catenin beta-1. To test this hypothesis, we analyzed the effects of ROBO4 on RAC1 activity by measuring active RAC1 (RAC1-GTP) using a G-LISA (Fig. 3a). Active RAC1 levels transiently increased 30 min after TNF treatment and then decreased at later time points in HUVECs transfected with control siRNA. In contrast, *ROBO4* siRNA induced RAC1 activation 240 and 480 min after the TNF treatment, in addition to the activation at 30 min. The activated RAC1 levels at 240 min and 480 min in cells treated with *ROBO4* siRNA were significantly greater than those in cells treated with control siRNA. These results indicated that ROBO4 specifically suppresses TNF-induced activation of RAC1 at later time points.

To investigate the effects of ROBO4 on RAC1 downstream signaling, we analyzed JNK phosphorylation (Fig. 3b). *ROBO4* siRNA enhanced TNF-induced phosphorylation of JNK. The increased JNK phosphorylation induced by *ROBO4* siRNA was inhibited by an RAC1 inhibitor (NSC23766), indicating that ROBO4 suppressed RAC1-JNK signaling (Fig. 3c). In addition, SP600125, an inhibitor of RAC1 and JNK, suppressed *PTGS2* upregulation induced by *ROBO4* siRNA (Fig. 3d, e). Taken together, these results indicate that ROBO4 suppresses TNF-induced PTGS2 expression by inhibiting RAC1-JNK signaling.

### ROBO4-mediated RAC1 inhibition suppresses vascular hyperpermeability

**Fig. 4:**
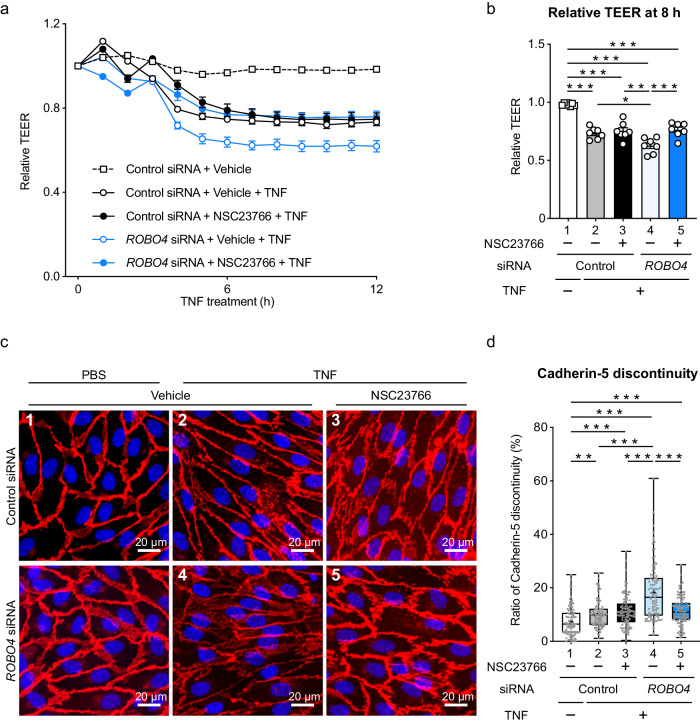
ROBO4 suppresses TNF-induced endothelial hyperpermeability by inhibiting RAC1. **a** Effect of a RAC1 inhibitor on *ROBO4* knockdown-induced endothelial hyperpermeability. HUVECs were treated with siRNA and NCS23766 (100 μM), stimulated with TNF, and used for the measurement for TEER. Relative TEER was calculated. **b** Relative TEER at 8 h after TNF stimulation. **c** Effect of the RAC1 inhibitor on cadherin-5 localization in *ROBO4*-knockdown ECs. Scale bars indicate 20 μm. **d** Quantification of cadherin-5 discontinuity. One hundred cells in four images obtained from two independent experiments are analyzed. Boxes show first, second (median), and third quartiles, and whiskers show maximum and minimum ratios of cadherin-5 discontinuity. Data are expressed as the mean ± standard error of the mean (**a**, **b**). *P < 0.05, **P < 0.01, and ***P < 0.001, calculated using one-way analysis of variance followed by Tukey’s test (**b**) or Kruskal–Wallis’s test (**d**). Non-specified *P* values in the graph are not significant.

Although ROBO4 has been shown to suppress endothelial hyperpermeability induced by TNF, its underlying mechanism remains unclear^15^. To investigate whether ROBO4 also suppresses permeability by inhibiting RAC1 activation, we analyzed the permeability of HUVEC monolayers treated with siRNA and the RAC1 inhibitor by measuring TEER (Fig. 4a, b and Supplementary Fig. S3). In HUVECs treated with control siRNA, TNF decreased TEER and induced hyperpermeability, whereas the RAC1 inhibitor did not affect TEER. In contrast, *ROBO4* siRNA enhanced the TNF-induced decrease in the TEER in HUVECs, while the RAC1 inhibitor suppressed this TEER decrease. These results indicated that ROBO4 suppresses TNF-induced endothelial hyperpermeability by suppressing RAC1.

We analyzed the effect of RAC1 on cadherin-5 (also known as VE-cadherin) localization (Fig. 4c, d). TNF treatment induced EC elongation, as reported previously^41^. In cells treated with control siRNA, TNF treatment induced discontinuous cadherin-5 localization (at cell junctions), which remained unaffected by the RAC1 inhibitor. In contrast, *ROBO4* siRNA increased discontinuous cadherin-5 localization at cell junctions; this phenomenon was inhibited by the RAC1 inhibitor. Taken together, ROBO4 suppresses TNF-mediated endothelial hyperpermeability by inhibiting RAC1 activation and stabilizing cadherin-5 localization at cell junctions.

### ROBO4 regulates RAC1 activity via TRAF7 and IQGAP1 ubiquitination

**Fig. 5:**
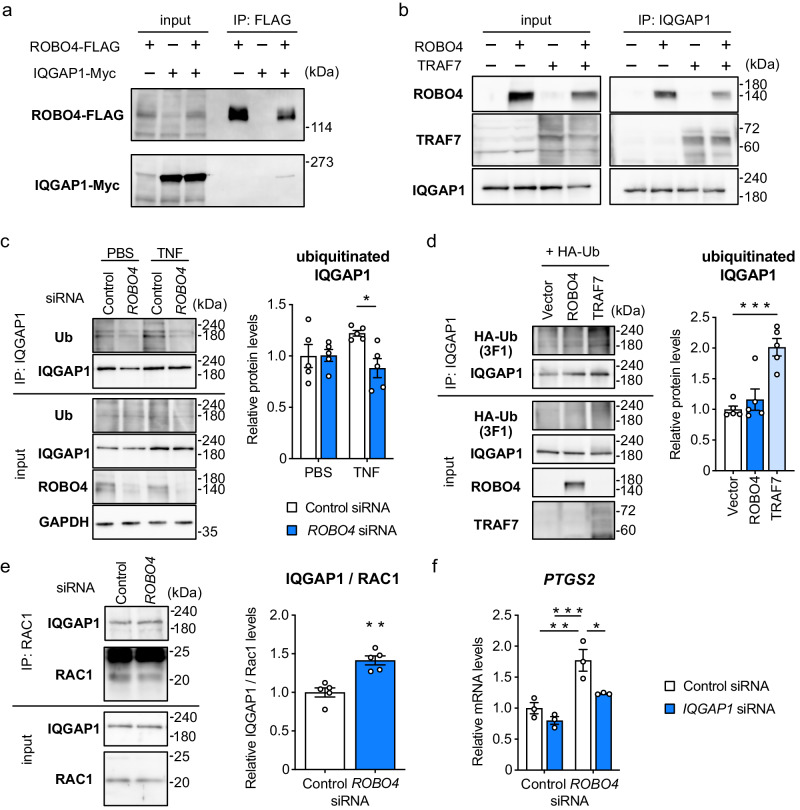
ROBO4 promotes IQGAP1 ubiquitination via TRAF7 and suppresses PTGS2 expression. **a** Interaction between ROBO4 and IQGAP1. FLAG-tagged ROBO4 and Myc-tagged IQGAP1 were expressed in COS-7 cells, and lysates were immunoprecipitated with an anti-FLAG antibody. Precipitates were analyzed by western blotting. **b** Interaction between IQGAP1 and TRAF7 in the presence and absence of ROBO4. HEK293 cells were transfected with ROBO4 and TRAF7 expression vectors, and lysates were immunoprecipitated with an antibody against endogenous IQGAP1. Precipitates were analyzed by western blotting. **c** ROBO4-mediated IQGAP1 ubiquitination in ECs. HUVECs were treated with siRNA and TNF, and lysates were immunoprecipitated with anti-IQGAP1 antibody. Precipitates were analyzed using antibodies against ubiquitin, IQGAP1, and ROBO4. Immunoblots were quantified using ImageJ software and relative ubiquitinated IQGAP1 levels were calculated (*n* = 5). **d** IQGAP1 ubiquitination was enhanced by TRAF7, but not by ROBO4. HEK293 cells were transfected with expression vectors for ROBO4 or TRAF7, and lysates were immunoprecipitated with the anti-IQGAP1 antibody. Precipitates were analyzed by western blotting. Ubiquitinated IQGAP1 was quantified using ImageJ (*n* = 5). **e** Interaction between IQGAP1 and RAC1 in the presence or absence of ROBO4. HUVECs were transfected with control and *ROBO4* siRNA, and lysates were immunoprecipitated with an antibody for endogenous RAC1. Precipitates were analyzed by western blotting. **f** Contribution of IQGAP1 to *ROBO4* knockdown-mediated *PTGS2* expression. (*n* = 3). Data are expressed as the mean ± standard error of the mean (**c**–**f**). *P < 0.05, **P < 0.01, and ***P < 0.001, *P* values were calculated with a two-way analysis of variance followed by Tukey’s test (**c**, **e**) or one-way analysis of variance followed by Dunnett’s test (**d**). Non-specified *P* values in the graph are not significant.

To investigate the mechanism through which ROBO4 regulates RAC1 activity, we immunoprecipitated ROBO4-interacting proteins from HUVECs expressing FLAG-tagged ROBO4. Mass spectrometry analysis of the proteins identified the Ras GTPase-activating-like protein IQGAP1 (Table S1). The interaction between ROBO4 and IQGAP1 was confirmed by a co-immunoprecipitation assay using COS-7 cells expressing ROBO4 and IQGAP1 (Fig. 5a).

IQGAP1 is known to interact with active RAC1 (RAC1-GTP) and sustains its activity^42,43^. However, ubiquitination of IQGAP1 inhibits this interaction and inactivates RAC1^44^. Based on this information, we hypothesized that ROBO4 and TRAF7 ubiquitinate IQGAP1 via direct interactions and promote dissociation between IQGAP1 and RAC1 to inactivate RAC1. To test this hypothesis, we first analyzed the interaction among ROBO4, TRAF7, and IQGAP1 (Fig. 5b). Immunoprecipitation assays demonstrated that IQGAP1 interacted with ROBO4 and TRAF7 in HEK293 cells (wherein the endogenous expression levels of ROBO4 and TRAF7 are low), suggesting a ternary complex consisting of IQGAP1, ROBO4, and TRAF7. We analyzed the effect of ROBO4 on IQGAP1 ubiquitination (Fig. 5c). *ROBO4* siRNA decreased IQGAP1 ubiquitination in TNF-treated HUVECs but not in untreated cells. In addition, overexpression of TRAF7, but not that of ROBO4, induced ubiquitination of IQGAP1 in HEK293 cells (Fig. 5d), indicating that TRAF7 is the main regulator of IQGAP1 ubiquitination and that ROBO4 functions as an enhancer of this process. Additionally, *ROBO4*-knockdown enhanced the interaction between IQGAP1 and RAC1 (Fig. 5e). Taken together, our results showed that ROBO4 promoted IQGAP1 ubiquitination via TRAF7 and induced dissociation of IQGAP1 from RAC1.

Finally, we analyzed the effects of IQGAP1 on *ROBO4*-knockdown-induced *PTGS2* upregulation in TNF-treated ECs (Fig. 5f). *IQGAP1*-knockdown inhibited PTGS2 expression in cells transfected with *ROBO4* siRNA, but not in those transfected with control siRNA, indicating that ROBO4 suppressed PTGS2 expression via IQGAP1. Taken together, our results showed that ROBO4 and TRAF7 promoted IQGAP1 ubiquitination, suppressed RAC1 activity by inducing dissociation of IQGAP1 from RAC1, and suppressed PTGS2 expression in TNF-treated ECs.

### Robo4 ameliorates inflammatory diseases in mouse models

Since our in vitro results revealed that ROBO4 suppressed PTGS2 expression in ECs, we investigated the roles of Robo4 in mouse models of Ptgs2-associated inflammatory diseases, including arthritis^45^, inflammatory edema^46^, and pain^47^.

**Fig. 6:**
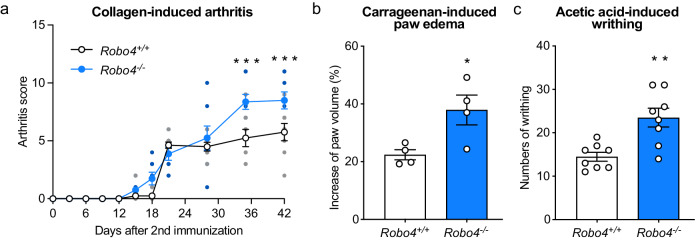
Robo4 ameliorates PTGS2-associated inflammatory disease in mouse models. **a** Collagen-induced arthritis models using *Robo4*^+/+^ and *Robo4*^−^^/^^−^ mice (*n* = 8). Clinical scores after the second immunization were evaluated at each time point. **b** Carrageenan-induced paw edema models using *Robo4*^+/+^ and *Robo4*^−^^/^^−^ mice (*n* = 4). Paw volume of the right hind limb before and 4 h after injection of 1% λ-carrageenan was measured and the increase in paw volumes was calculated. **c** Inflammatory pain models using *Robo4*^+/+^ and *Robo4*^−^^/^^−^ mice (*n* = 8). The mice were intraperitoneally injected with 0.6% acetic acid, and the number of writhing behaviors was counted for 30 min. Data are expressed as the mean ± standard error of the mean (**a**–**c**). *P < 0.05, **P < 0.01, and ***P < 0.001, *P* values were calculated with a one-way analysis of variance followed by Bonferroni test (**a**), and Welch’s *t*-test (**b**, **c**). Non-specified *P* values in the graph are not significant.

In the arthritis model, Robo4-deficient (*Robo4*^−/^^−^) and wild-type (*Robo4*^+/+^) mice were subcutaneously injected with type II collagen with Freund’s complete adjuvant twice, and their rheumatoid arthritis scores were subsequently assessed according to the evaluation criteria (Fig. 6a and Table S4). The scores in *Robo4*^+/+^ and *Robo4*^−^^/^^−^ mice almost reached a plateau at 21 days and 35 days after injection, respectively. The scores at 35 days and 42 days in *Robo4*^−^^/^^−^ mice were significantly higher than those in *Robo4*^+/+^ mice. During this assay, body weight did not differ between *Robo4*^+/+^ and *Robo4*^−^^/^^−^ mice (Supplementary Fig. S4). In the Ptgs2-associated edema model, carrageenin solution was injected to the right hind paw plantar of *Robo4*^+/+^ and *Robo4*^−^^/^^−^ mice. *Robo4*^−^^/^^−^ mice showed enhanced hind limb edema as compared to *Robo4*^+/+^ mice (Fig. 6b). In the Ptgs2-associated pain model, *Robo4*^-/-^ mice were intraperitoneally injected with acetic acid, and their writhing behaviors, characterized by contraction of the abdomen and extension of the trunk and hind limbs, we counted over 30 min. The number of writhing behaviors observed in *Robo4*^-/-^ mice was higher than that in *Robo4*^+/+^ mice (Fig. 6c). Taken together, these results indicated that Robo4 suppressed Ptgs2-associated inflammatory conditions such as arthritis, edema, and pain.

## Discussion

**Fig. 7:**
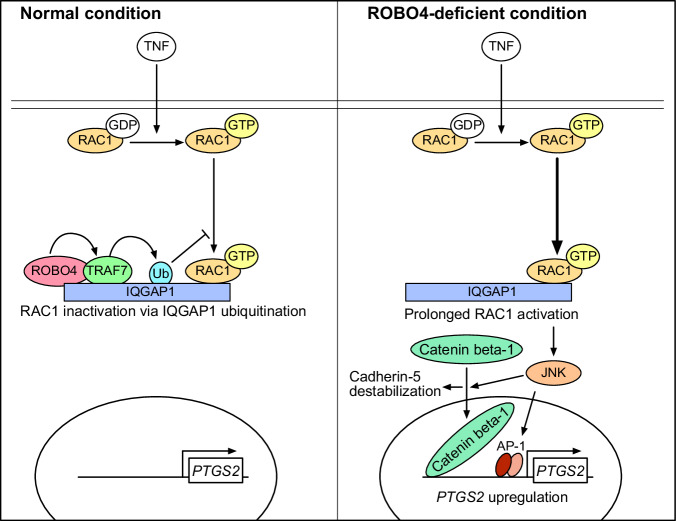
A regulatory model for ROBO4-mediated suppression of PTGS2 expression and endothelial permeability via RAC1 inhibition. In ECs treated with TNF, a ROBO4–TRAF7 complex ubiquitinates IQGAP1 and induces RAC1 dissociation from IQGAP1, which leads to RAC1 inactivation. However, in ROBO4-deficient ECs, RAC1 is activated because of decreased ubiquitination of IQGAP1. The prolonged RAC1 activation induces PTGS2 expression and endothelial hyperpermeability partially through JNK-AP1/catenin beta-1 signaling. Consistently, ROBO4 deficiency exacerbates PTGS2-associated inflammatory diseases in mice.

ROBO4 suppresses multiple diseases by stabilizing ECs in mice. In particular, ROBO4 suppresses severe infectious and inflammatory diseases, including COVID-19 and sepsis, by decreasing vascular permeability. However, it was unclear whether Robo4 suppresses diseases only by suppressing vascular permeability. In this study, we demonstrated that ROBO4 decreases PTGS2 expression and ameliorates PTGS2-associated inflammatory diseases in mice. Mechanistically, ROBO4 decreased TNF-induced PTGS2 expression by suppressing the RAC1–JNK–AP1/catenin beta-1 axis. In addition, ROBO4 suppressed TNF-induced endothelial hyperpermeability by inhibiting RAC1. ROBO4 suppressed prolonged RAC1 activation by interacting with IQGAP1 and TRAF7, and promoted IQGAP1 ubiquitination, which led to RAC1 inactivation by dissociating IQGAP1 from RAC1^44^. Thus, we successfully revealed the unknown ROBO4 function as a regulator of endothelial PTGS2 expression and the mechanism whereby ROBO4 suppresses RAC1 activation (Fig. 7). Our findings indicated that ROBO4 suppressed inflammatory responses through multiple actions, including suppression of vascular permeability and PTGS2 expression and ROBO4 can be a potential therapeutic target in various inflammatory diseases.

In this study, we found that ROBO4 suppressed TNF-induced PTGS2 expression by inhibiting RAC1. Interestingly, in the G-LISA analysis, ROBO4 specifically suppressed RAC1 activation at later time points after TNF stimulation but did not affect activation at the early time point. This suggests that ROBO4 suppresses prolonged RAC1 activation and terminates TNF-induced RAC1 signaling. In addition, ROBO4-mediated RAC1 inhibition suppressed endothelial hyperpermeability. However, previous reports have indicated that ROBO4 regulates RAC1 activity both positively and negatively. For example, ROBO4 promoted RAC1 activation in non-ECs and porcine aortic ECs^21^, whereas ROBO4 suppressed RAC1 activation in human microvascular ECs^16^, indicating that ROBO4-mediated RAC1 regulation is cell type- and species-dependent. The mechanism of ROBO4-mediated RAC1 regulation found in this study may support our understanding of the controversial results reported by previous studies.

A detailed mechanistic analysis of ROBO4-mediated RAC1 suppression demonstrated that ROBO4 binds to IQGAP1 and promotes its ubiquitination via TRAF7. We identified TRAF7 as an E3 ubiquitin ligase for IQGAP1 in ECs. A previous study using non-ECs indicated that IQGAP1 specifically binds to active RAC1 and sustains its activity^48^, while the ubiquitination of IQGAP1 dissociates active RAC1 from IQGAP1, thereby inactivating RAC1^44^. In this study, we found that IQGAP1 was ubiquitinated by ROBO4 and TRAF7 in ECs and regulated RAC1 activity. This mechanism explains how ROBO4 terminates prolonged RAC1 activation.

Although we demonstrated that ROBO4 suppressed endothelial hyperpermeability by inhibiting RAC1, we found that the RAC1 inhibitor did not completely suppress TNF-induced hyperpermeability. In contrast, our previous study demonstrated that overexpression of ROBO4 or TRAF7 suppressed TNF-induced hyperpermeability^15^. This suggested that ROBO4 and TRAF7 suppressed endothelial hyperpermeability not only by inhibiting RAC1 activity but also by regulating that of other permeability-related proteins. TRAF7 regulates vascular permeability by activating MEKK3 through SCRIB^49,50^, suggesting the existence of a potential RAC1-independent mechanism by which TRAF7 suppresses vascular permeability. Further studies are needed to completely understand the regulation of vascular permeability by ROBO4 and TRAF7.

In this study, we used a relatively high concentration of TNF to effectively demonstrate the effects of ROBO4 on the inflammatory response. However, it is crucial to perform experiments using TNF concentrations that are more reflective of those observed in inflammatory diseases in vivo is important. We demonstrated that ROBO4 suppressed PTGS2 expression in ECs treated with lower TNF concentrations (Supplementary Fig. S2a). Although this result suggests that ROBO4 function is unlikely to be influenced by TNF concentration, further comprehensive investigations with lower TNF concentrations that are observed in carrageenan and acetic acid-injection models^33,51^ are required. In addition to *PTGS2*, our RNA-seq analysis suggested that *TGFB2* is another candidate for the ROBO4-regulated gene (Fig. 1d, e and Supplementary Fig. S5). Since TGFB2 is involved in the regulation of inflammatory responses and diseases, it should be important to investigate how ROBO4 regulates TGFB2 expression in inflammatory ECs.

In summary, we demonstrated that ROBO4 suppresses inflammatory diseases by inhibiting PTGS2 expression and vascular hyperpermeability by suppressing prolonged RAC1 activation. These multiple ROBO4 functions explain why ROBO4 effectively suppresses a wide range of diseases associated with the endothelial inflammatory response. In addition, our recent findings indicate that small molecules that enhance ROBO4 expression suppress vascular permeability and mortality in mouse endotoxemia and COVID-19 models^22^. This result suggests that ROBO4-increasing compounds may ameliorate inflammatory diseases by suppressing PTGS2 expression. Therefore, these results highlight ROBO4 as an important therapeutic target for various diseases.

### Supplementary information

Supplementary Information

Description of Additional Supplementary Files

Supplementary Data 1

Supplementary Data 2

Reporting Summary

## Data Availability

All other data are available from the corresponding authors or other sources on reasonable request. Uncropped blot images are provided in Supplementary Figs. S6–8. RNA-seq data have been deposited to the GEO and are available at the accession number GSE231460. Source data for the graphs and mass spectrometry analysis are provided in Supplementary Datas 1 and 2, respectively. The raw mass spectrometry data generated in the core facility of Osaka University in 2014 is not available and cannot be deposited in an external repository.
