# Supplementary material for: Endothelial ROBO4 suppresses PTGS2/COX-2 expression and inflammatory diseases
Source: Commun Biol. 2024 May 18;7:599. doi: 10.1038/s42003-024-06317-z (PMC11102558; doi:10.1038/s42003-024-06317-z)
Supplement: Supplementary file 1 — Supplementary Information [file 42003_2024_6317_MOESM1_ESM.pdf]

Endothelial ROBO4 suppresses PTGS2/COX-2 expression and inflammatory diseases

Masato Tanaka[1]*, Keisuke Shirakura[1]*, Yui Takayama[1], Miki Matsui[1], Yukio Watanabe[2], Takuya Yamamoto[2,3,4], Junya Takahashi[1], Shota Tanaka[1], Nobumasa Hino[1], Takefumi Doi[1], Masanori Obana[1,5], Yasushi Fujio[1,5], Kazuo Takayama[2,6†], Yoshiaki Okada[1,5†]

[1]Graduate School of Pharmaceutical Sciences, Osaka University, Osaka, Japan
[2]Center for iPS Cell Research and Application (CiRA), Kyoto University, Kyoto, Japan
[3]Medical-risk Avoidance based on iPS Cells Team, RIKEN Center for Advanced Intelligence Project (AIP), Kyoto, Japan
[4]Institute for the Advanced Study of Human Biology (WPI-ASHBi), Kyoto University, Kyoto, Japan
[5]Center for Infectious Disease Education and Research (CiDER), Osaka University, Osaka, Japan
[6]AMED-CREST, Japan Agency for Medical Research and Development (AMED), Tokyo, Japan

[†]Correspondence to:

Yoshiaki Okada, Ph.D., Graduate School of Pharmaceutical Sciences, Osaka University, 1-6 Yamadaoka, Suita, Osaka 565-0871, Japan; Tel and Fax: +81-6-6879-8164

Kazuo Takayama, Ph.D., Center for iPS Cell Research and Application (CiRA), Kyoto University, Shogoin Kawaharacho 53, Sakyo-ku, Kyoto 606-8507, Japan; Tel and Fax: +81-75-366-7362, +81-75-366-7098

* These authors contributed equally to this work

Supplemental Figures

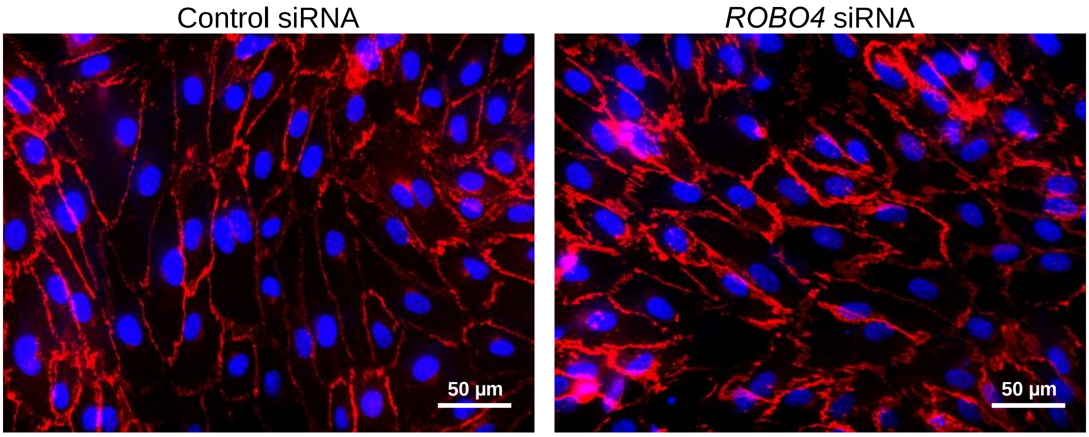

Supplementary Figure S1. Cadherin-5 staining using HUVECs treated with siRNA and TNF. HUVECs were transfected with siRNA, and then treated with TNF for 6 h. Subsequently, immunofluorescence staining was performed to visualize Cadherin-5 expression. These culture conditions are the same as those used for RNA-seq analysis.

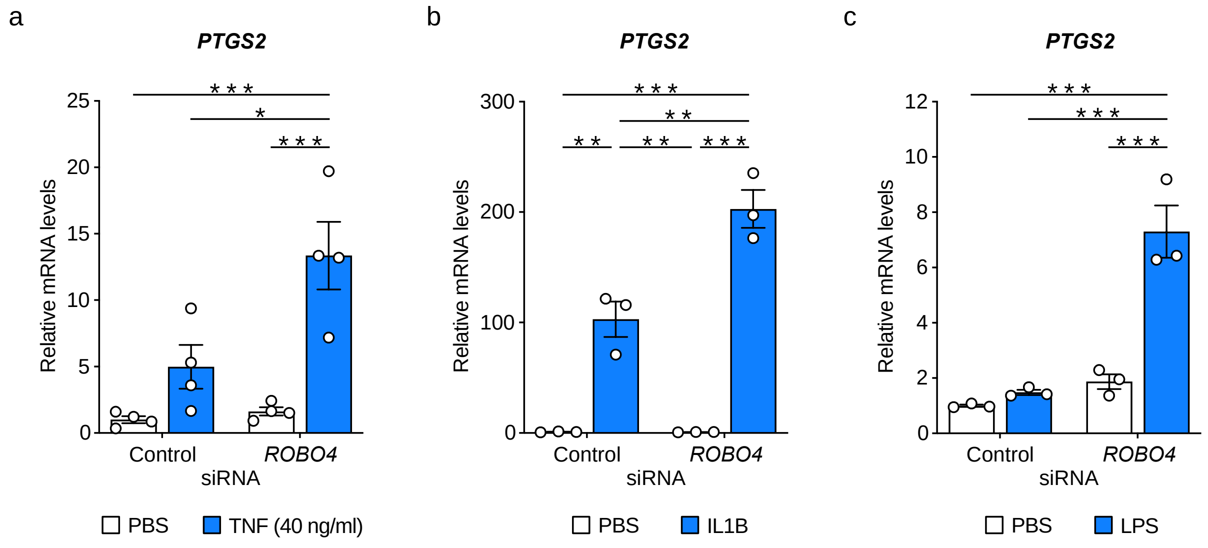

Supplementary Figure S2. ROBO4 suppresses PTGS2 expression induced by IL1B and LPS. (a–c) Effect of ROBO4 on PTGS2 expression in endothelial cells treated with TNF (40 ng/ml) for 6 h (a), IL1B for 8 h (b), or LPS (c) for 8 h. Expression of *PTGS2* and *GAPDH* mRNA was measured using qRT-PCR (n = 3 or 4). Data are expressed as the mean ± standard error of the mean. *P < 0.05, **P < 0.01, ***P < 0.001, calculated via two-way analysis of variance followed by Tukey's test (a–c). Non specified p values in the graph are not significant.

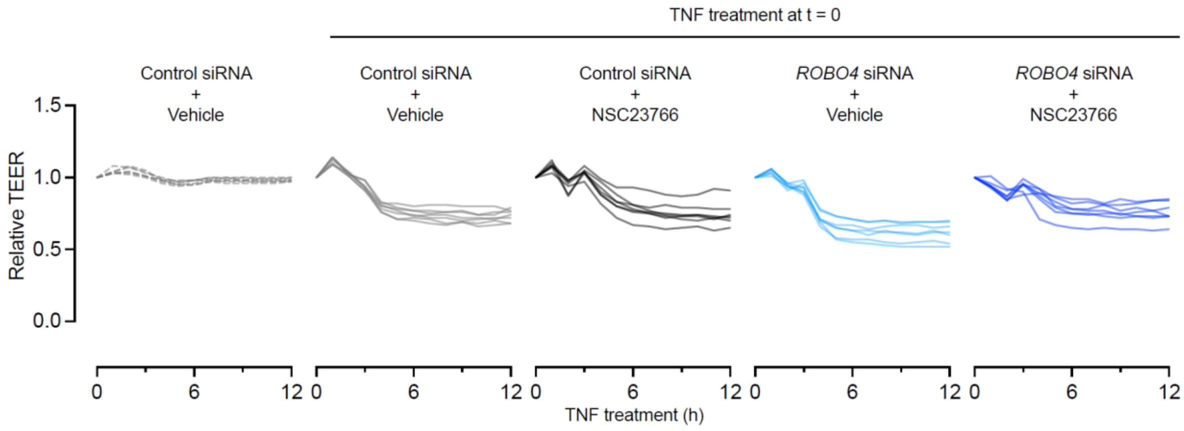

Supplementary Figure S3. Relative TEER values for each sample in Figure 4a

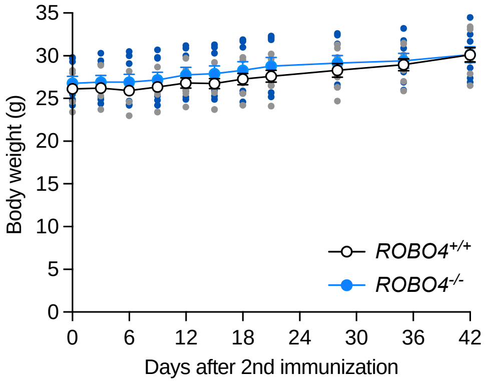

Supplementary Figure S4. Mouse body weights in collagen-induced arthritis models. Body weights in *Robo4*$^{+/+}$ and *Robo4*$^{-/-}$ mice (n = 8) were measured. Data are expressed as the mean ± standard error of the mean.

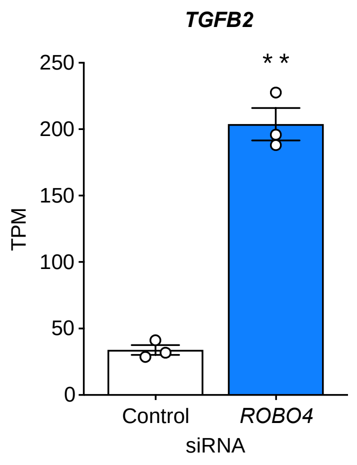

Supplementary Figure S5. Effect of ROBO4 on TGFB2 expression in TNF-treated ECs. Transcripts per million (TPM) value of TGFB2 in HUVECs treated with TNF and siRNA for Control or *ROBO4*. Data are expressed as the mean ± standard error of the mean. **P <0.01; P values were calculated using Welch's t-test.

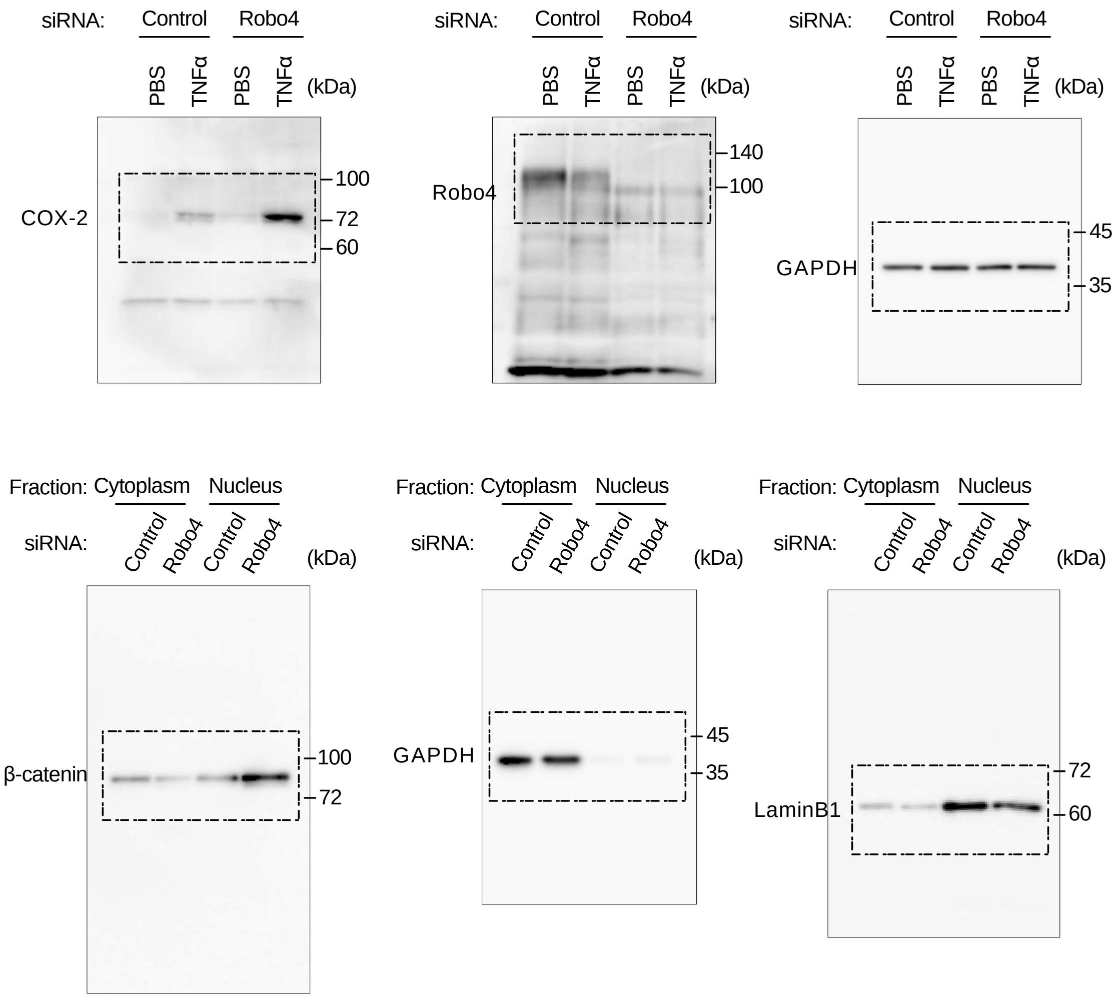

Supplementary Figure S6. Uncropped blot images for Figure 2.

Figure 3b

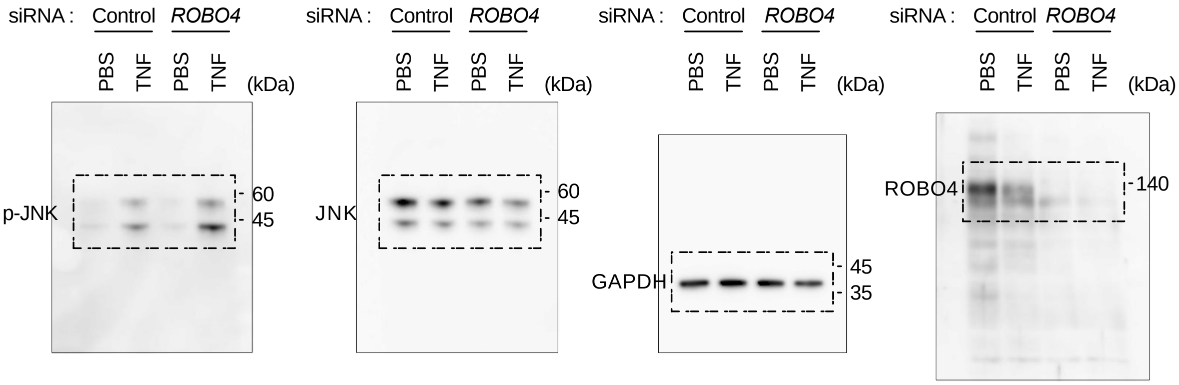

Figure 3c

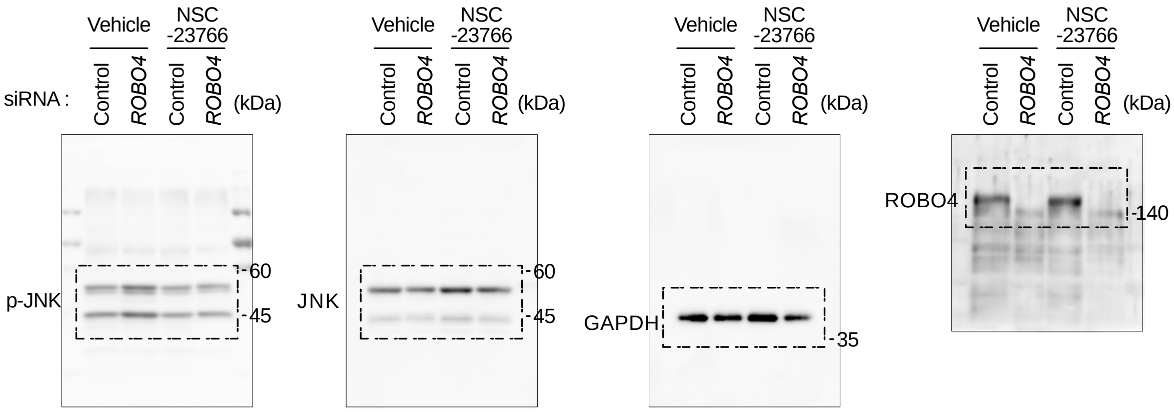

Supplementary Figure S7. Uncropped blot images for Figure 3.

Figure 5a

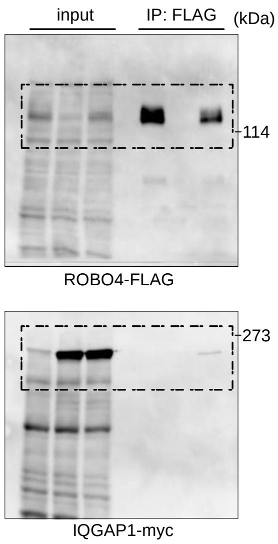

Figure 5b

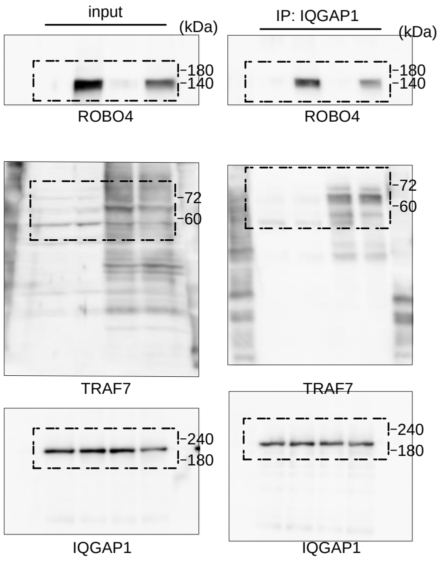

Figure 5c

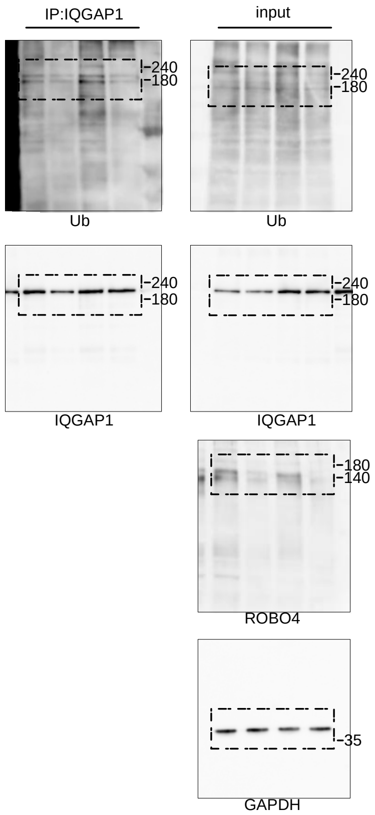

Figure 5d

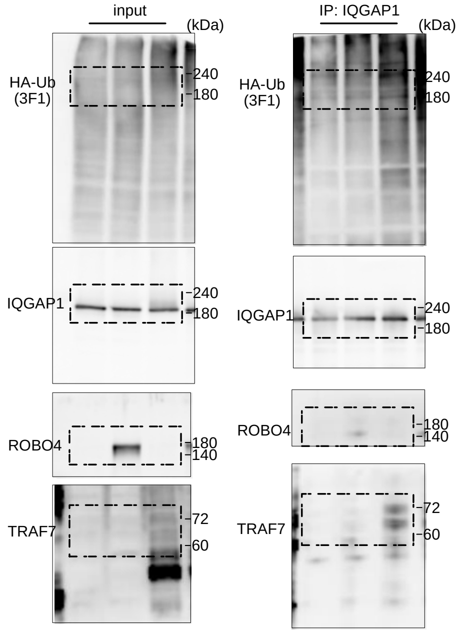

Figure 5e

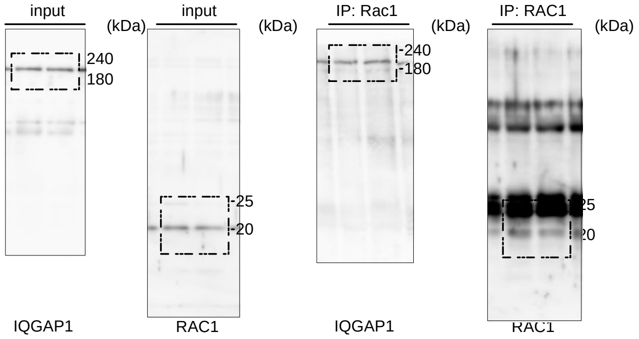

Supplementary Figure S8. Uncropped blot images for Figure 5.

Supplemental Tables

Supplementary Table S1. Unique peptides identified by mass spectrometry

Identified unique peptides
(K)LGNFFSPK(V)
(K)LGLAPQIQDLYGK(V)
(R)LAAVALINAAIQK(G)
(R)ILAIGLINEALDEGDAQK(T)
(K)FLSAIVSSVDK(I)
(K)TEVSLTLTNK(F)

Supplementary Table S2. Primer and siRNA sequences

Real-time RT-PCR		5'-3'
ROBO4	Forward	TTATGGCTCCCTCATCGCTG
	Reverse	GAGGCTGTCTGAGCTGGAAC
GAPDH	Forward	TGGAGTCCACTGGCGTCTTC
	Reverse	GGCTGTTGTCATACTTCTCATGGT
PTGS2	Forward	GGTCTGGTGCCTGGTCTGAT
	Reverse	TGTTTAAGCACATCGCATACTCTGT
Construction of expression vectors		
IQGAP1-myc	Forward	CTTTCCAAGGTTTCACGGCTTC
	Reverse	CATTGCTCGAGTAACTTCCCGATGA
HA-Ub	Forward	CACACAGGTACCACCATGTACCCATACGATGTTCCGG ATTACGCTCAGATC
	Reverse	CACACACTCGAGTCAGCCACCCCTCAGGCGCAGGACC AGGTGCAGGGTCG
Sequences of siRNA		
ROBO4		GCGAGGGCUCCUUAGCCAATT
CTNNB1		TAAGAATTGAGTAATGGTGTA
IQGAP1		AAGGAGACGTCAGAACGTGGC

Supplementary Table S3. Antibodies

Antibody name	Clone name	Company	Catalog number
β-catenin	E-5	Santa Cruz Biotechnology	sc-7963
Cox2	D5H5	Cell Singling Technology	12282
M2 FLAG tag		Sigma Aldrich	F1804
GAPDH	1E6D9	Proteintech	60004-1-Ig
HA tag	3F10	Roche	ROAHAHA
IQGAP1	H-109	Santa Cruz Biotechnology	sc-10792
JNK		Cell Singling Technology	9252
Phospho-JNK	81E11	Cell Singling Technology	4668
LaminB1	3G10G12	Proteintech	66095-1-Ig
Myc tag	9B11	Cell Singling Technology	2276
Robo4		R&D Systems	AF2366
TRAF7	H-300	Santa Cruz Biotechnology	sc-98963
Mono- and poly-ubiquitinylated conjugates	FK2	Enzo	BML-PW8810-0100
Rac1		Invitrogen	PA1-091
Rac1		Sigma-Aldrich	05-389

Supplementary Table S4. Arthritis scores

Score	Characteristics
0	Normal
1	Slight swelling in one joint
2	Swelling in multiple joints or slight swelling in entire paw
3	Severe swelling in entire paw
4	Severe swelling in entire paw and joint rigidity

Supplementary Table S5. P-values

Figure	Statistical method	Comparison	P-value	Summary
Figure 1F	Unpaired t-test with Welch's correction	Control siRNA vs. ROBO4 siRNA	0.002	**
Figure 2A	Tukey's test	Contol siRNA:PBS vs. Contol siRNA:TNF	0.0044	**
		Contol siRNA:PBS vs. ROBO4 siRNA:PBS	0.9098	
		Contol siRNA:PBS vs. ROBO4 siRNA:TNF	<0.0001	***
		Contol siRNA:TNF vs. ROBO4 siRNA:PBS	0.0139	*
		Contol siRNA:TNF vs. ROBO4 siRNA:TNF	0.0003	***
		ROBO4 siRNA:PBS vs. ROBO4 siRNA:TNF	<0.0001	***
Figure 2B	Tukey's test	Control siRNA:PBS vs. Control siRNA:TNF	0.0495	*
		Control siRNA:PBS vs. ROBO4 siRNA:PBS	0.8032	
		Control siRNA:PBS vs. ROBO4 siRNA:TNF	0.0001	***
		Control siRNA:TNF vs. ROBO4 siRNA:PBS	0.2172	
		Control siRNA:TNF vs. ROBO4 siRNA:TNF	0.0134	*
		ROBO4 siRNA:PBS vs. ROBO4 siRNA:TNF	0.0004	***
Figure 2C	Tukey's test	Control siRNA-vehicle vs. ROBO4 siRNA-vehicle	0.9637	
		Control siRNA-vehicle vs. Control siRNA-SR11302	<0.0001	***
		Control siRNA-vehicle vs. ROBO4 siRNA-SR11302	0.9967	
		ROBO4 siRNA-vehicle vs. Control siRNA-SR11302	<0.0001	***
		ROBO4 siRNA-vehicle vs. ROBO4 siRNA-SR11302	0.9022	
		Control siRNA-SR11302 vs. ROBO4 siRNA-SR11302	0.0001	***

Figure 2D	Tukey's test	Control siRNA:Control siRNA vs. Control siRNA:CTNNB1 siRNA	0.0019	**
		Control siRNA:Control siRNA vs. ROBO4 siRNA:Control siRNA	<0.0001	***
		Control siRNA:Control siRNA vs. ROBO4 siRNA:CTNNB1 siRNA	<0.0001	***
		Control siRNA:CTNNB1 siRNA vs. ROBO4 siRNA:Control siRNA	0.0003	***
		Control siRNA:CTNNB1 siRNA vs. ROBO4 siRNA:CTNNB1 siRNA	0.5561	
		ROBO4 siRNA:Control siRNA vs. ROBO4 siRNA:CTNNB1 siRNA	0.0074	**
Figure 2E	Unpaired t-test with Welch's correction	Control siRNA vs. ROBO4 siRNA	0.0017	**
Figure 3A	Boferroni's test	at 0 min	>0.9999	
		at 15 min	>0.9999	
		at 30 min	0.2052	
		at 60 min	>0.9999	
		at 240 min	0.0032	**
		at 280 min	0.0001	**
Figure 3B	Tukey's test	Contol siRNA:PBS vs. Contol siRNA:TNF	0.0285	*
		Contol siRNA:PBS vs. ROBO4 siRNA:PBS	0.5734	
		Contol siRNA:PBS vs. ROBO4 siRNA:TNF	<0.0001	***
		Contol siRNA:TNF vs. ROBO4 siRNA:PBS	0.2897	
		Contol siRNA:TNF vs. ROBO4 siRNA:TNF	0.008	**
		ROBO4 siRNA:PBS vs. ROBO4 siRNA:TNF	0.0002	***
Figure 3C	Tukey's test	Control siRNA:vehicle vs. Control siRNA:NSC23766	0.2273	
		Control siRNA:vehicle vs. ROBO4 siRNA:vehicle	0.0003	***
		Control siRNA:vehicle vs. ROBO4 siRNA:NSC23766	0.3041	
		Control siRNA:NSC23766 vs. ROBO4 siRNA:vehicle	<0.0001	***

		Control siRNA:NSC23766 vs. ROBO4 siRNA:NSC23766	0.0102	*
		ROBO4 siRNA:vehicle vs. ROBO4 siRNA:NSC23766	0.0059	**
Figure 3D	Tukey's test	Control siRNA:vehicle vs. Control siRNA:NSC23766	0.5719	
		Control siRNA:vehicle vs. ROBO4 siRNA:vehicle	<0.0001	***
		Control siRNA:vehicle vs. ROBO4 siRNA:NSC23766	0.0088	**
		Control siRNA:NSC23766 vs. ROBO4 siRNA:vehicle	<0.0001	***
		Control siRNA:NSC23766 vs. ROBO4 siRNA:NSC23766	0.1114	
		ROBO4 siRNA:vehicle vs. ROBO4 siRNA:NSC23766	0.0046	**
Figure 3E	Tukey's test	Control siRNA:vehicle vs. Control siRNA:SP600125	0.0133	*
		Control siRNA:vehicle vs. ROBO4 siRNA:vehicle	0.0013	**
		Control siRNA:vehicle vs. ROBO4 siRNA:SP600125	0.8654	
		Control siRNA:SP600125 vs. ROBO4 siRNA:vehicle	0.0001	***
		Control siRNA:SP600125 vs. ROBO4 siRNA:SP600125	0.0013	**
		ROBO4 siRNA:vehicle vs. ROBO4 siRNA:SP600125	0.0133	*
Figure 4B	Tukey's test	Control siRNA + PBS + vehicle vs. Control siRNA + TNF + vehicle	<0.0001	***
		Control siRNA + PBS + vehicle vs. Control siRNA + TNF + NSC23766	<0.0001	***
		Control siRNA + PBS + vehicle vs. ROBO4 siRNA + TNF + vehicle	<0.0001	***
		Control siRNA + PBS + vehicle vs. ROBO4 siRNA+ TNF + NSC23766	<0.0001	***
		Control siRNA + TNF + vehicle vs. Control siRNA + TNF + NSC23766	0.963	

		Control siRNA + TNF + vehicle vs. ROBO4 siRNA + TNF + vehicle	0.0131	*
		Control siRNA + TNF + vehicle vs. ROBO4 siRNA+ TNF + NSC23766	0.8608	
		Control siRNA + TNF + NSC23766 vs. ROBO4 siRNA + TNF + vehicle	0.0023	**
		Control siRNA + TNF + NSC23766 vs. ROBO4 siRNA+ TNF + NSC23766	0.9976	
		ROBO4 siRNA + TNF + vehicle vs. ROBO4 siRNA+ TNF + NSC23766	0.001	**
Figure 4D	Kruskal-Wallis's test	Control siRNA + PBS + vehicle vs. Control siRNA + TNF + vehicle	0.0053	**
		Control siRNA + PBS + vehicle vs. Control siRNA + TNF + NSC23766	<0.0001	***
		Control siRNA + PBS + vehicle vs. ROBO4 siRNA + TNF + vehicle	<0.0001	***
		Control siRNA + PBS + vehicle vs. ROBO4 siRNA+ TNF + NSC23766	<0.0001	***
		Control siRNA + TNF + vehicle vs. Control siRNA + TNF + NSC23766	>0.9999	
		Control siRNA + TNF + vehicle vs. ROBO4 siRNA + TNF + vehicle	<0.0001	***
		Control siRNA + TNF + vehicle vs. ROBO4 siRNA+ TNF + NSC23766	>0.9999	
		Control siRNA + TNF + NSC23766 vs. ROBO4 siRNA + TNF + vehicle	<0.0001	***
		Control siRNA + TNF + NSC23766 vs. ROBO4 siRNA+ TNF + NSC23766	>0.9999	
		ROBO4 siRNA + TNF + vehicle vs. ROBO4 siRNA+ TNF + NSC23766	0.0001	***

Figure 5C	Tukey's test	PBS:Control siRNA vs. PBS:ROBO4 siRNA	>0.9999	
		PBS:Control siRNA vs. TNF:Control siRNA	0.2424	
		PBS:Control siRNA vs. TNF:ROBO4 siRNA	0.7264	
		PBS:ROBO4 siRNA vs. TNF:Control siRNA	0.2617	
		PBS:ROBO4 siRNA vs. TNF:ROBO4 siRNA	0.6976	
		TNF:Control siRNA vs. TNF:ROBO4 siRNA	0.0376	*
Figure 5D	Dunnett's test	empty vector vs. ROBO4	0.6168	
		empty vector vs. TRAF7	0.0003	***
Figure 5E	Unpaired t-test with Welch's correction	Control siRNA vs. ROBO4 siRNA	0.001	**
Figure 5F	Tukey's test	Control siRNA:Control siRNA vs. Control siRNA:IQGAP1 siRNA	0.5549	
		Control siRNA:Control siRNA vs. ROBO4 siRNA:Control siRNA	0.0033	**
		Control siRNA:Control siRNA vs. ROBO4 siRNA:IQGAP1 siRNA	0.4297	
		Control siRNA:IQGAP1 siRNA vs. ROBO4 siRNA:Control siRNA	0.0007	***
		Control siRNA:IQGAP1 siRNA vs. ROBO4 siRNA:IQGAP1 siRNA	0.0704	
		ROBO4 siRNA:Control siRNA vs. ROBO4 siRNA:IQGAP1 siRNA	0.0258	*
Fiure 6A	Bonferroni's test	Day0	>0.9999	
		Day3	>0.9999	
		Day6	>0.9999	
		Day9	>0.9999	
		Day12	>0.9999	
		Day15	>0.9999	
		Day18	0.17	
		Day21	>0.9999	
		Day28	>0.9999	
		Day35	<0.0001	***
		Day42	0.0002	***
Figure 6B	Unpaired t-test	Robo4 +/+ vs. Robo4 -/-	0.0294	*

Figure 6C	Unpaired t-test with Welch's correction	Robo4 +/+ vs. Rob4 -/-	0.0036	**
