# Supplementary material for: Endothelial ROBO4 suppresses PTGS2/COX-2 expression and inflammatory diseases
Source: Commun Biol. 2024 May 18;7:599. doi: 10.1038/s42003-024-06317-z (PMC11102558; doi:10.1038/s42003-024-06317-z)
Supplement: Supplementary file 2 — Description of Additional Supplementary Files [file 42003_2024_6317_MOESM2_ESM.pdf]

Description of Additional Supplementary Files

File name: Supplementary Data 1

Description: Source data behind the graphs in the paper.

File name: Supplementary Data 2

Description: Source data for mass spectrometry analysis.
