# Supplementary material for: Endothelial ROBO4 suppresses PTGS2/COX-2 expression and inflammatory diseases
Source: Commun Biol. 2024 May 18;7:599. doi: 10.1038/s42003-024-06317-z (PMC11102558; doi:10.1038/s42003-024-06317-z)
Supplement: Supplementary file 5 — Reporting Summary [file 42003_2024_6317_MOESM5_ESM.pdf]

nature portfolio

Corresponding author(s): Yoshiaki Okada, Kazuo Takayama

Last updated by author(s): Mar 8, 2024

Reporting Summary

Nature Portfolio wishes to improve the reproducibility of the work that we publish. This form provides structure for consistency and transparency in reporting. For further information on Nature Portfolio policies, see our Editorial Policies and the Editorial Policy Checklist.

Statistics

For all statistical analyses, confirm that the following items are present in the figure legend, table legend, main text, or Methods section.

n/a	Confirmed	
☐	☒	The exact sample size (*n*) for each experimental group/condition, given as a discrete number and unit of measurement
☐	☒	A statement on whether measurements were taken from distinct samples or whether the same sample was measured repeatedly
☐	☒	The statistical test(s) used AND whether they are one- or two-sided<br>*Only common tests should be described solely by name; describe more complex techniques in the Methods section.*
☐	☒	A description of all covariates tested
☐	☒	A description of any assumptions or corrections, such as tests of normality and adjustment for multiple comparisons
☐	☒	A full description of the statistical parameters including central tendency (e.g. means) or other basic estimates (e.g. regression coefficient) AND variation (e.g. standard deviation) or associated estimates of uncertainty (e.g. confidence intervals)
☐	☒	For null hypothesis testing, the test statistic (e.g. *F*, *t*, *r*) with confidence intervals, effect sizes, degrees of freedom and *P* value noted<br>*Give P values as exact values whenever suitable.*
☒	☐	For Bayesian analysis, information on the choice of priors and Markov chain Monte Carlo settings
☒	☐	For hierarchical and complex designs, identification of the appropriate level for tests and full reporting of outcomes
☒	☐	Estimates of effect sizes (e.g. Cohen's *d*, Pearson's *r*), indicating how they were calculated

Our web collection on statistics for biologists contains articles on many of the points above.

Software and code

Policy information about availability of computer code

Data collection	ImageJ/Fiji version 1.54f
Data analysis	GraphPad Prism 10 for mac OS Version 10.2.0 (335)

For manuscripts utilizing custom algorithms or software that are central to the research but not yet described in published literature, software must be made available to editors and reviewers. We strongly encourage code deposition in a community repository (e.g. GitHub). See the Nature Portfolio guidelines for submitting code & software for further information.

Data

Policy information about availability of data

All manuscripts must include a data availability statement. This statement should provide the following information, where applicable:

- Accession codes, unique identifiers, or web links for publicly available datasets
- A description of any restrictions on data availability
- For clinical datasets or third party data, please ensure that the statement adheres to our policy

Raw data from this study were submitted under the Gene Expression Omnibus (GEO) (accession number GSE231460).

Research involving human participants, their data, or biological material

Policy information about studies with human participants or human data. See also policy information about sex, gender (identity/presentation), and sexual orientation and race, ethnicity and racism.

Reporting on sex and gender	n/a
Reporting on race, ethnicity, or other socially relevant groupings	n/a
Population characteristics	n/a
Recruitment	n/a
Ethics oversight	n/a

Note that full information on the approval of the study protocol must also be provided in the manuscript.

Field-specific reporting

Please select the one below that is the best fit for your research. If you are not sure, read the appropriate sections before making your selection.

☒ Life sciences ☐ Behavioural & social sciences ☐ Ecological, evolutionary & environmental sciences

For a reference copy of the document with all sections, see nature.com/documents/nr-reporting-summary-flat.pdf

Life sciences study design

All studies must disclose on these points even when the disclosure is negative.

Sample size	Each assay was conducted using a sample size that was the same as or larger than the typical sample size shown in several previous papers.
Data exclusions	No data were excluded from the analyzes.
Replication	Each assay was performed multiple times to ensure reproducibility.
Randomization	No grouping was performed according to specific criteria in each experiment.
Blinding	The animal experiments were conducted by researchers who were unaware of the research details.

Reporting for specific materials, systems and methods

We require information from authors about some types of materials, experimental systems and methods used in many studies. Here, indicate whether each material, system or method listed is relevant to your study. If you are not sure if a list item applies to your research, read the appropriate section before selecting a response.

Materials & experimental systems

n/a	Involved in the study	
☐	☒	Antibodies
☐	☒	Eukaryotic cell lines
☒	☐	Palaeontology and archaeology
☐	☒	Animals and other organisms
☒	☐	Clinical data
☒	☐	Dual use research of concern
☒	☐	Plants

Methods

n/a	Involved in the study	
☒	☐	ChIP-seq
☒	☐	Flow cytometry
☒	☐	MRI-based neuroimaging

Antibodies

Antibodies used	β-catenin (E-5), Santa Cruz Biotechnology, #sc-7963<br>Cox2 (D5H5) Cell Singling Technology, #12282<br>M2 FLAG tag, Sigma Aldrich, #F1804<br>GAPDH (1E6D9), Proteintech, #60004-1-Ig

HA tag (3F10), Roche, #ROAHAHA
IQGAP1 (H-109), Santa Cruz Biotechnology, #sc-10792
JNK, Cell Singling Technology, #9252
Phospho-JNK (81E11), Cell Singling Technology, #4668
LaminB1 (3G10G12), Proteintech #66095-1-Ig
Myc tag (9B11), Cell Singling Technology, #2276
Robo4, R&D Systems, #AF2366
TRAF7 (H-300), Santa Cruz Biotechnology, #sc-98963
mono- and poly-ubiquitinylated conjugates (FK2), Enzo, #BML-PW8810-0100
Rac1 (PA1-091), Invitrogen, #PA1-091
Rac1 (23A8), Sigma-Aldrich, #05-389

Validation

Validation Information of the antibodies were obtained from manufacturer's web site and previous papers. Antibodies were also validated by the experiments shown in the figures.

Eukaryotic cell lines

Policy information about cell lines and Sex and Gender in Research

Cell line source(s)

Primary human umbilical vein endothelial cells (HUVECs) were purchased from Lonza. Human embryonic kidney cells (HEK293) and African Green monkey SV40-transfected kidney fibroblast cells (COS-7 cells) were obtained from ATCC.

Authentication

The purchased cells are strictly controlled to ensure that there are no mix-ups. We also confirmed the morphology and function of each cell.

Mycoplasma contamination

We confirmed that the cells were free of mycoplasma infection using a commercially available kit.

Commonly misidentified lines
(See ICLAC register)

n/a

Animals and other research organisms

Policy information about studies involving animals; ARRIVE guidelines recommended for reporting animal research, and Sex and Gender in Research

Laboratory animals

Robo4+/+ and Robo4-/- mice (C57/BL6N back ground, male, 11-13 weeks old)

Wild animals

n/a

Reporting on sex

In line with the general idea of avoiding instability of experimental results due to the estrous cycle, we created an inflammation model using male mice.

Field-collected samples

n/a

Ethics oversight

The animal studies were approved by the ethics committees of Charles River Laboratories Japan, Inc. (approval numbers 1358, 1359, and 1579) and Osaka University (approval number 28-15).

Note that full information on the approval of the study protocol must also be provided in the manuscript.

Plants

Seed stocks

n/a

Novel plant genotypes

n/a

Authentication

n/a
